# Diazinon Interrupts Ovarian Steroidogenic Acute Regulatory (*StAR*) Gene Transcription in Gonadotropin-Stimulated Rat Model

**Published:** 2018

**Authors:** Asma Siavashpour, Younes Ghasemi, Bahman Khalvati, Fereshteh Jeivad, Negar Azarpira, Hossein Niknahad

**Affiliations:** a *Department of Pharmacology and Toxicology, School of Pharmacy, Shiraz University of Medical Sciences, Shiraz, Iran. *; b *Department of Pharmaceutical Biotechnology, School of Pharmacy, Shiraz University of Medical Sciences, Shiraz, Iran. *; c *Pharmaceutical Sciences Research Center, Shiraz University of Medical Sciences, Shiraz, Iran.*; d *Medicinal Plants Research Center, Yasuj Univercity of Medical Sciences, Yasuj, Iran. *; e *Department of Toxicology and Pharmacology, School of Pharmacy, Tehran University of Medical Sciences, Tehran, Iran. *; f *Transplant Research Center, Shiraz University of Medical Sciences,Shiraz, Iran.*

**Keywords:** Diazinon, *Corpus Luteum*, StAR gene, Progesterone, Real time PCR, Time dependent

## Abstract

Organophosphate pesticides are considered as endocrine disruptors that interfere with reproductive functions. The *corpus luteum* (CL) is a transient endocrine gland that produces progesterone, a crucial hormone for a successful beginning and maintenance of pregnancy*.* Steroidogenic acute regulatory protein (*StAR*) facilitates the rate-limiting transfer of cholesterol from the outer mitochondrial membrane to the inner organelle membranes. We investigated the effect of Diazinon (DZN), an organophosphate, on *StAR* mRNA expression by Sybergreen Real Time-PCR in a time-dependent manner in luteal phase. Fifty immature female Wistar rats (24-day-old) were injected with a single injection of Pregnant mare’s Serum Gonadotropin (PMSG) followed by a single injection of human Chorionic Gonadotropin (hCG), 48 h later. Then, DZN was administered in a single dose (70 mg/kg bw, I.P), controls received only the vehicle, 12 h post-hCG injection. Ovaries were collected in 4 h. intervals from 8 to 24 h post-hCG injection. Then, hCG stimulation transcript levels of *StAR* gene were significantly altered in the hormone-stimulated rats following DZN treatment. In addition, histological study showed that the CL diameter in DZN-treated group was smaller than control group (*p* = 0.000). Our findings suggest that the critical step in the function of CL is disrupted by DZN and may correlates with female reproductive damage.

## Introduction

 Organophosphate (OP) compounds are a group of widely used insecticides in agriculture (1, 2), Also they are used in the urban setting to protect home foundations from termites and prevent the spread of West Nile virus carried by mosquitoes (3). Diazinon (DZN; 0,0-diethyl-0-[2-isopropyl-6-methyl-4-pyramidinyl] phosphorothioate) after malathion is one of the most commonly used OPs on agricultural crops in developing countries; mainly known by their neurotoxic effects due to the inhibition of acetylcholinesterase enzyme (AChE) (4, 5). In general, OPCs have a greater acute toxicity than other pesticides like organochlorine compounds (6). And since they are endocrine disruptors that documented to be associated with increased incidences of reproductive dysfunction (7). Although this reproductive dysfunction is characterized by spermatogenic disturbances, semen quality deterioration, testicular degeneration and hormone imbalances, the mechanisms involved in OPs-induced infertility remain unclear (7-13). 

 There is increasing evidence suggesting that the decreasing trends in fertility rates not only in many industrialized countries, but also developing countries are now so dramatic as a global phenomenon. Public concern over the possibility that fertility may be at risk from exposure to pesticides is increasing (14, 15). Since recent epidemiological and experimental studies suggest that OPs pesticides as endocrine disrupter may increase the risk of early pregnancy loss in women under certain occupational exposure situations (16). However, a few of studies have showed that OPs may contribute to endocrine disruption effects in wildlife. A number of studies suggest that OPs including chlorpyrifos, Dimethoate can reduce serum progesterone hormone level (6, 17). Also, it was reported that carbaryl carbamate insecticide could increase spontaneous abortion rate in wives of exposed workers (18). Some studies indicated that DZN and malathion used in commercial formulation could be toxic to *in-vitro* fertilization and embryo development (19). They can induce infertility, spontaneous abortions, and physical malformations in human and animals (20-22). 

 The *corpus luteum* (CL) exerts an essential role for the establishment and maintenance of pregnancy in human (23) and full pregnancy in rats by sustaining progesterone secretion (24). The process of luteinization is associated with up-regulation of steroidogenic acute regulatory (StAR) protein in luteinized granulose and theca cells (25).

 StAR protein, a phosphoprotein expressed in steroidogenic cells, is essential for sterol translocation process from the outer to inner mitochondrial membrane in response to tropic hormones (LH and hCG), the rate limiting step in CL steroidogenesis (26, 27), StAR mRNA and protein expression has been reported in the several species CL, including the mouse (28), rat (29), rabbit (30), dog (31) and human. StAR mRNA expression and protein levels are regulated within the CL throughout the luteal phase, playing a key role in controlling luteal progesterone production during the development and demise of CL (32).

 Exposure to pesticides may involve large segments of population which include agriculture workers and their wives, communities living in areas with intensive agricultural activity, besides the general population that may be exposed through home application of pesticides or via residues in the soil, water bodies, vegetables and other food products (33, 34). Studies on OPCs effects on the female reproductive system and the hormone level are very rare, although hormonal balance plays a main role in female reproductive function. However, the U.S. Environmental Protection Agency (EPA) phased-out almost all residential and other similar uses of DZN the USA at the end of 2004, they continue to be widely used throughout the commercial agricultural industry (35, 36) and in developing countries (37). Therefore, this study was designed to examine the diazinon effect, as a model of OPs on StAR mRNA expression in the ovary of gonadotropin-stimulated immature rat model.

## Experimental

*Chemicals*

 Diazinon (DZN) from Merck Co.(Germany) (99% purity**), **pregnant mare’s serum gonadotrophin (PMSG) from Intervet Inc.(Germany), human chorionic gonadotropin (hCG) from Intervet Inc.(Germany), RNX plus solution from Sinaclon (Iran), cDNA first strand synthesis kit from Fermentase (Germany), and SYBR Green Premix 2X from Takara (Japan) were used in this study.

*Animals*

 Immature Wistar female rats were obtained from the Pastor Institute (Experimental Animal Center, Shiraz, Iran) and held in our laboratory. The animals were fed a standard laboratory diet and water ad libitum and housed in a temperature-controlled room (22 ± 2 °C) in cages with a 12 h light-dark cycle, relative humidity of 50–55%, then all studies were begun when the animals were 24 days old (36-38 g). All procedures used were approved by the University Animal Care and Use Committee, Shiraz University of Medical Sciences. The procedures were performed in accordance with institution guidelines for animal care and use. 

*Experimental design*

 Fifty Immature 24-day-old Wistar rats were randomly divided into 5 time groups; four time groups comprised of 10 rats, 5 as control and 5 as DZN treatment for each time point. One time group was considered without injection before ovulation time (8 h post-hCG). All the animals employed in this study received intra-peritoneal (I.P) injection with PMSG (15 IU) to stimulate follicular maturation. Forty-eight h later, the rats were injected with hCG (15 IU, I.P) to induce ovulation process. DZN was dissolved in corn oil to consistent absorption and administered in a single dose (70 mg/kg body weight, I.P); control animals received only the vehicle under the same condition; at 12 h post-hCG injection (around ovulation time). The LD50 of DZN in female rats is 300 mg/kg body weight (38), which was taken as the reference value. The reason for selecting a dose of 70 mg/kg bw in the present experiment is selected based on pilot study in our laboratory to determine its sub lethal dose that caused toxicity to the animals and simultaneously did not cause mortality of the animals.

 The rats were sacrificed by spinal dislocation at defined time points at 4-h intervals from 8 to 24 h post-hCG injection. The ovaries were rapidly removed, washed in a cold saline solution, snap-frozen in liquid nitrogen and then stored at -80 °C for RNA extraction.

*Histological analysis*

 The ovaries were dissected and fixed in Bouin’s fixative for histological examination. The fixed tissue was cut into 5-7-μm thick sections and stained with hematoxylin and eosin.

 For each ovary, at least six independent sections were selected, and the total number of CL and Graafian follicles was counted. At least 100 CL and follicles (FL) in different stages were counted and the fraction of FL/CL was calculated for each case.

 The follicles were classified as Secondary when they had more than two layers of granulosa cells and labeled as antral when the follicle contained fluid. In the Graafian follicle, the oocyte occupied an excentric position and the antral cavity was filled with fluid. 

 The size of CL was measured by computer-assisted morphometric program (Olysia, Olumpus).

*Preparation of total RNA and cDNA syntetase*

 Total RNA was extracted from approximately 100 mg of ovary tissue by RNX plus solution (Sinaclon, Iran) in a clean RNase-free tube. Concentration and purity of RNA were quantified by NanoDrop ND-100 spectrophotometer (Thermo Scientific, Waltham, MA, USA) at 260 nm and 280 nm.

RNA was converted to cDNA after treating with DNase I. Reverse transcription of RNA was done in a final volume of 20 μL by using cDNA first strand synthesis kit (Fermentase, Germany) by oligo (dT) primer

*Quantitative Real-Time PCR*

 The sequence of the gene was obtained from Gen Bank and the primers were used in previous studies (39, 40). The sequences of the primers are shown in Table 1. For real-time quantitative PCR, 500 ng of RT product was used in whole volume of 25 μL containing 7.5 μL of SYBR Green Premix 2X (Takara, Shiga, Japan) and 10 PM of mix primer. Thermocycling conditions were; heat hold at 94 °C for 1 min followed by 40 cycles of denaturation at 94 °C for 20 sec, annealing and extention at 61 °C for 30 sec. We used the ΔΔ*CT *method for determination of relative StAR gene expression. The *Ct *of sample was compared with the *Ct *of its internal control (β-actin). Real-time PCR was done with a BioradBiosystemsIQ5 detection system. All reactions were done in duplicate. Specificity of PCR reaction was double-confirmed by electrophoresis and melting curve analysis.

*Statistics*

Statistical analysis of data was carried out using SPSS (version 18) software. The results are expressed as mean ± SE of five experiments. One-way analysis of variance (ANOVA) and Bonferroni multiple comparison tests were used to test the difference between groups. *P *< 0.05 for comparison between study groups was taken as statistically significant.

## Results

*Corpus luteum formation*

**Figure 1:**
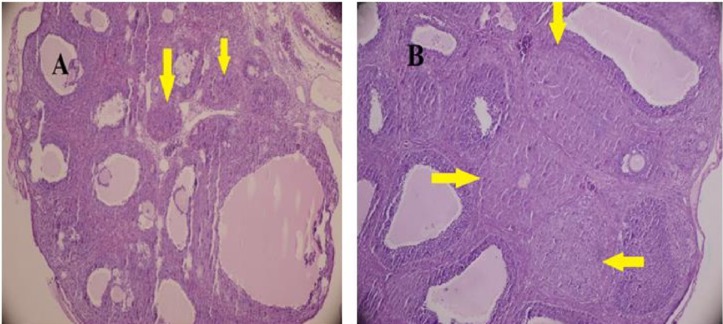
Histological feature of gonadotropin-induced rat ovaries after treatment with either DZN (A) or corn oil (control) (B ). The ovaries were collected at 24 h post-hCG injection and tissue sections were stained with hematoxylin and eosin. (H&E staining;**× **400 ). The pointer shows CL. CL was smaller in diameter in DZN group, (diameter: control, 0.62 ± 0.08; DZN group, 0.31 ± 0.12 μm; *P *= 0.000

The gonadotropin-primed immature female rat is a well-studied and accepted animal model for ovulatory function. The administration, a combination PMSG and hCG in high doses induces the ovarian hyperstimulation (41). Histological analysis of the ovaries indicated the follicular development and CL formation (Figure 1). CL, growing follicles and Graafian follicles were found in all ovaries. However, CL was smaller in diameter in test group (diameter: control, 0.62 ± 0.08; test animals, 0.31 ± 0.12 μm; *P* = 0.000).The fraction of FL/CL did not have significant differences in both groups (4.35 in DZN-treated groups , 4.37 in control, *P* = 0.9)

**Table 1:** Primer sequences used for quantitative real-time PCR.

**Gene**	**Primers**	**Primer Sequence**	**Expected size in base pair**
β-actin	Forward Reverse	5'-ACC AAC TGG GAC GAT ATG GAG AAG A-3' 5'-TAC GAC CAG AGG CAT ACA GGG ACA A-3'	214
StAR	Forward Reverse	5'-GCA GCA ACT GCA GCA CTA CCA CAG AA-3' 5'-GTA TGC CCA AGG CCT TTT GCA TAG CTT-3'	160

**Figure 2:**
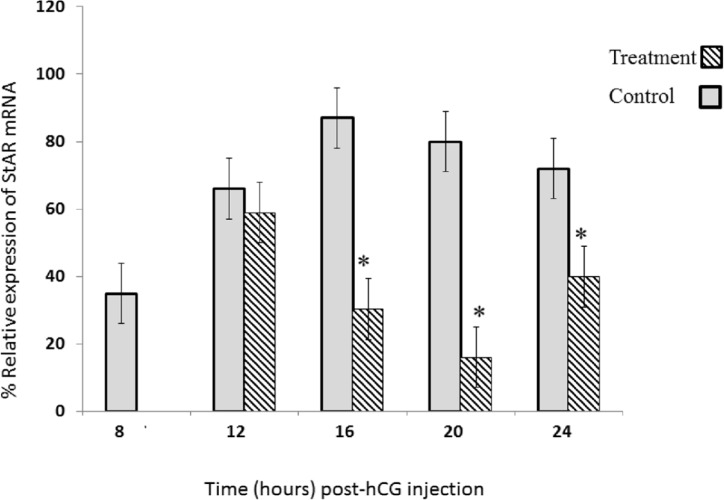
DZN Effect on StAR mRNA expression; in gonadotropin-induced rat ovary in a time-dependent pattern. Sybergreen real-time assay was performed for StAR mRNA analysis in gonadotropin-induced rat ovary after treatment with either DZN or corn oil (control). Relative levels of mRNA are expressed as the ratio of the target genes relative to β-actin in each sample. Values are means ± S.E. of groups of five rats. ^*^Significantly different from respective control group, *P* < 0.05

*StAR expression after hormonal stimulation*

StAR mRNA expression was assessed using Sybergreen Real Time RT-PCR. This analysis reveals significant changes in the levels of StAR mRNA in response to PMSG and hCG. First, we compared the relative transcript levels of StAR mRNA in ovary extract that prepared time dependently trend following the administration of PMSG and hCG to prepubertal rats (Figure 2).

The results showed that the StAR expression was rapidly high after ovulation and during luteinization (at 12 and 16 h) in control group (Figure 2). The peak of StAR mRNA was observed at 16 h post-hCG administration in control rats. Interestingly, the level of StAR mRNA markedly dropped to 7% of its maximal value at 20 h post-hCG (Figure 2). 

*StAR expression after diazinon administration*

While, a significant decrease was not observed in StAR mRNA expression 12 h post hCG (Figure 2), its expression was significantly decreased at 16, 20, and 24 h post hCG, in DZN-treatment group compared to the control group. Maximum inhibitory effect of DZN on StAR mRNA expression was at 20 h post hCG. The values of StAR expression were 35%, 55.5%, and 20% of the control group at 16, 20, and 24 h, respectively(Figure 2).

## Discussion

Previous studies showed that OP and carbamate insecticides suppressed* in-vitro* progesterone synthesis via inhibiting StAR mRNA expression at a dose-dependent manner (6, 17), but there is limited evidence about its *in-vivo* effects and female genus. To our knowledge, the present study for the first time has examined DZN effect as OPs on CL formation and StAR mRNA expression in the ovary of gonadotropin-stimulated immature rat model in a time-dependent manner.

Histological data of the present study did not show a significant difference (*P* = 0.9) in the fraction of FL/CL between control (4.37) and DZN-treated groups (4.35) at 24 h post-hCG. It is conceivable because of folliculogenesis and ovulation that occurred after gonadotropin administration could not be affected by DZN. Interestingly, in the DZN-treated group, the CL was formed but was smaller in diameter than control group. This suggests that DZN suppress the normal development of CL. 

In addition, we observed that CL formation is related to a dramatic increase in StAR mRNA expression in gonadotropin-stimulated immature rats. In the other hand, DZN administration decreased StAR mRNA expression at 16, 20 and 24 h post hCG compared to the control group during luteinization that would be one of reasons for small CL diameter in DZN-treated group.

StAR mRNA and protein expression are increased in a time-dependent manner, in the early and middle of the luteal cycle that is necessary for CL formation and function. It is positively correlated with progesterone concentrations throughout the early and mid-luteal phase (42-44). OPs could effect on StAR mRNA and CL formation by different pathway including LH, prolactin and estradiol level (45). LH pulses during the luteal phase are critical for the development and function of luteinized cell (46, 47). In male genus, it is established that organophosphates could have effect on hypothalamic-pituitary axis and cause decreased LH level (48-50) which most likely can be attributed to a reduction in StAR transcription (45). In addition, prolactin potentiate both LH and estrogen receptor expression and in this way has positive effect on StAR gene transcription (46, 51). OPs increased hypothalamic acetylcholine and dopamine levels (52-54) and so have directly negative effect on prolactin secretion. 

This study indicated that decreased StAR mRNA expression due to DZN exposure could disrupt CL development and decrease its Steroidogenesis ability during early critical period in luteal phase which cause impaired fertility. StAR expression is the time-related changes biomarker. Its disruption may represent the first event in the sequence of time-related changes that underlie pesticide-induced toxicity and lead to disturbances at the Steroidogenesis as a major function for fertility. 

Environmental pollutant potently influence StAR gene transcription (55) and today increasing trend in different pesticide usage concomitant with other environmental pollutant would have effect on StAR mRNA and protein level in synergism manner. Therefore, this research suggests studies on interaction between mixtures of chemical for their Steroidogenesis inhibition ability. 

## References

[1] Harris W, Sachana M, Flaskos J, Hargreaves AJ (2009). Proteomic analysis of differentiating neuroblastoma cells treated with sub-lethal neurite inhibitory concentrations of diazinon: identification of novel biomarkers of effect. Toxicol. Appl. Pharmacol.

[2] Kitamura K, Maruyama K, Hamano S, Kishi T, Kawakami T, Takahashi Y, Onodera S (2014). Effect of hypochlorite oxidation on cholinesterase-inhibition assay of acetonitrile extracts from fruits and vegetables for monitoring traces of organophosphate pesticides. J. Toxicol. Sci.

[3] Ghafour-Rashidi Z, Dermenaki-Farahani E, Aliahmadi A, Esmaily H, Mohammadirad A, Ostad SN, Abdollahi M (2007). Protection by cAMP and cGMP phosphodiesterase inhibitors of diazinon-induced hyperglycemia and oxidative/nitrosative stress in rat Langerhans islets cells: Molecular evidence for involvement of non-cholinergic mechanisms. Pestic. Biochem. Physiol.

[4] Hariri AT, Moallem SA, Mahmoudi M, Memar B, Hosseinzadeh H (2010). Sub-acute effects of diazinon on biochemical indices and specific biomarkers in rats: Protective effects of crocin and safranal. Food Chem. Toxicol.

[5] Verma SK, Raheja G, Gill KD (2009). Role of muscarinic signal transduction and CREB phosphorylation in dichlorvos-induced memory deficits in rats: an acetylcholine independent mechanism. Toxicology.

[6] Walsh LP, Webster DR, Stocco DM (2000). Dimethoate inhibits steroidogenesis by disrupting transcription of the steroidogenic acute regulatory (StAR) gene. J. Endocrinol.

[7] Manabe M, Kanda S, Fukunaga K, Tsubura A, Nishiyama T (2006). Evaluation of the estrogenic activities of some pesticides and their combinations using MtT/Se cell proliferation assay. Int. J. Hyg. Environ. Health.

[8] Pina-Guzman B, Solis-Heredia MJ, Quintanilla-Vega B (2005). Diazinon alters sperm chromatin structure in mice by phosphorylating nuclear protamines. Toxicol. Appl. Pharmacol.

[9] Narayana K, Prashanthi N, Nayanatara A, Kumar HH, Abhilash K, Bairy KL (2005). Effects of methyl parathion (o,o-dimethyl o-4-nitrophenyl phosphorothioate) on rat sperm morphology and sperm count, but not fertility, are associated with decreased ascorbic acid level in the testis. Mutat. Res.

[10] Salazar-Arredondo E, de Jesus Solis-Heredia M, Rojas-Garcia E, Hernandez-Ochoa I, Quintanilla-Vega B (2008). Sperm chromatin alteration and DNA damage by methyl-parathion, chlorpyrifos and diazinon and their oxon metabolites in human spermatozoa. Reprod. Toxicol.

[11] Liu J, Park ES, Jo M (2009). Runt-related transcription factor 1 regulates luteinized hormone-induced prostaglandin-endoperoxide synthase 2 expression in rat periovulatory granulosa cells. Endocrinology.

[12] Dutta AL, Sahu CR (2013). Emblica officinalis Garten fruits extract ameliorates reproductive injury and oxidative testicular toxicity induced by chlorpyrifos in male rats. SpringerPlus.

[13] Volk M, Jaklic H, Zorn B, Peterlin B (2011). Association between male infertility and genetic variability at the PON1/2 and GSTM1/T1 gene loci. Reprod. Biomed. Online.

[14] Clementi M, Tiboni GM, Causin R, La Rocca C, Maranghi F, Raffagnato F, Tenconi R (2008). Pesticides and fertility: an epidemiological study in Northeast Italy and review of the literature. Reprod. Toxicol.

[15] Liu F, Liu WN, Zhao QX, Han MM (2013). Study on environmental and psychological risk factors for female infertility. Zhonghua lao dong wei sheng zhi ye bing za zhi.

[16] Clementi M, Causin R, Marzocchi C, Mantovani A, Tenconi R (2007). A study of the impact of agricultural pesticide use on the prevalence of birth defects in northeast Italy. Reprod. Toxicol.

[17] Hong X, Qu J, Wang Y, Sun H, Song L, Wang S, Wang X (2007). Study on the mechanism of trichlorfon-induced inhibition of progesterone synthesis in mouse leydig tumor cells (MLTC-1). Toxicology.

[18] Cheng S, Chen J, Qiu Y, Hong X, Xia Y, Feng T, Liu J, Zhang Z, Wang X (2006). Carbaryl inhibits basal and FSH-induced progesterone biosynthesis of primary human granulosa-lutein cells. Toxicology.

[19] Ducolomb Y, Casas E, Valdez A, Gonzalez G, Altamirano-Lozano M, Betancourt M (2009). In-vitro effect of malathion and diazinon on oocytes fertilization and embryo development in porcine. Cell Biol. Toxicol.

[20] Nishi K, Hundal SS (2013). Chlorpyrifos induced toxicity in reproductive organs of female Wistar rats. Food Chem. Toxicol.

[21] Mahadevaswami MP, Kaliwal BB (2004). Evaluation of dimethoate toxicity on pregnancy in albino mice. J. Basic. Clin. Physiol. Pharmacol.

[22] Jafarzadeh M, Nasri Nasrabadi Z, Sheikhazadi A, Abbaspour A, Vasigh S, Yousefinejad V, Marashi, SM (2013). Is there a role for progesterone in the management of acute organophosphate poisoning during pregnancy? Med. Hypotheses.

[23] Devoto L, Kohen P, Munoz A, Strauss JF 3rd (2009). Human corpus luteum physiology and the luteal-phase dysfunction associated with ovarian stimulation. Reprod. Biomed. Online.

[24] Goyeneche AA, Deis RP, Gibori G, Telleria CM (2003). Progesterone promotes survival of the rat corpus luteum in the absence of cognate receptors. Biol. Reprod.

[25] Morita Y, Wada-Hiraike O, Yano T, Shirane A, Hirano M, Hiraike H, Koyama S, Oishi H, Yoshino O, Miyamoto Y, Sone K, Oda K, Nakagawa S, Tsutsui K, Taketani Y (2012). Resveratrol promotes expression of SIRT1 and StAR in rat ovarian granulosa cells: an implicative role of SIRT1 in the ovary. Reprod. Biol. Endocrinol.

[26] Devoto L, Fuentes A, Kohen P, Cespedes P, Palomino A, Pommer R, Munoz A, Strauss JF 3rd (2009). The human corpus luteum: life cycle and function in natural cycles. Fertil. Steril.

[27] Jiang YF, Tsui KH, Wang PH, Lin CW, Wang JY, Hsu MC, Chen YC, Chiu CH (2011). Hypoxia regulates cell proliferation and steroidogenesis through protein kinase A signaling in bovine corpus luteum. Anim. Reprod. Sci.

[28] Clark BJ, Stocco DM (1995). Expression of the steroidogenic acute regulatory (StAR) protein: a novel LH-induced mitochondrial protein required for the acute regulation of steroidogenesis in mouse Leydig tumor cells. Endocr. Res.

[29] Sandhoff TW, Hales DB, Hales KH, McLean MP (1998). Transcriptional regulation of the rat steroidogenic acute regulatory protein gene by steroidogenic factor 1. Endocrinol.

[30] Townson DH, Wang XJ, Keyes PL, Kostyo JL, Stocco DM (1996). Expression of the steroidogenic acute regulatory protein in the corpus luteum of the rabbit: dependence upon the luteotropic hormone, estradiol-17 beta. Biol. Reprod.

[31] Kowalewski MP, Hoffmann B (2008). Molecular cloning and expression of StAR protein in the canine corpus luteum during dioestrus. Exp. Clin. Endocrinol. Diabetes.

[32] Devoto L, Kohen P, Gonzalez RR, Castro O, Retamales I, Vega M, Carvallo P, Christenson LK, Strauss JF 3rd (2001). Expression of steroidogenic acute regulatory protein in the human corpus luteum throughout the luteal phase. J. Clin. Endocrinol. Metab.

[33] Yehia MA, El-Banna SG, Okab AB (2007). Diazinon toxicity affects histophysiological and biochemical parameters in rabbits. Exp. Toxicol. Pathol.

[34] Abdou HM, ElMazoudy RH (2010). Oxidative damage, hyperlipidemia and histological alterations of cardiac and skeletal muscles induced by different doses of diazinon in female rats. J. Hazard. Mater.

[35] Morgan MK, Wilson NK, Chuang JC (2014). Exposures of 129 preschool children to organochlorines, organophosphates, pyrethroids, and acid herbicides at their homes and daycares in North Carolina. Int. J. Environ. Res. Public. Health.

[36] Rush T, Liu XQ, Hjelmhaug J, Lobner D (2010). Mechanisms of chlorpyrifos and diazinon induced neurotoxicity in cortical culture. Neuroscience.

[37] Banerjee I, Tripathi S, Roy AS (2012). Clinico-epidemiological characteristics of patients presenting with organophosphorus poisoning. N Am. J. Med. Sci.

[38] Gokcimen A, Gulle K, Demirin H, Bayram D, Kocak A, Altuntas I (2007). Effects of diazinon at different doses on rat liver and pancreas tissues. Pestic Biochem Physiol.

[39] Zhao D, Xue H, Artemenko I, Jefcoate C (2005). Novel signaling stimulated by arsenite increases cholesterol metabolism through increases in unphosphorylated steroidogenic acute regulatory (StAR) protein. Mol. Cell. Endocrinol.

[40] Z Hosaka Y, Ishibashi M, Wakamatsu Ji, Uehara M, Nishimura T (2012). Myostatin regulates proliferation and extracellular matrix mRNA expression in NIH3T3 fibroblasts. Biomed. Res.

[41] Liu J, Yang Y, Yang Y, Zhang Y, Liu W (2011). Disrupting effects of bifenthrin on ovulatory gene expression and prostaglandin synthesis in rat ovarian granulosa cells. Toxicol.

[42] Devoto L, Vega M, Kohen P, Castro O, Carvallo P, Palomino A (2002). Molecular regulation of progesterone secretion by the human corpus luteum throughout the menstrual cycle. J. Reprod. Immunol.

[43] Care AS, Diener KR, Jasper MJ, Brown HM, Ingman WV, Robertson SA (2013). Macrophages regulate corpus luteum development during embryo implantation in mice. J. Clin. Invest.

[44] Sertedaki A, Dracopoulou M, Voutetakis A, Stefanaki K, Rontogianni D, Magiakou AM, Kanaka-Gantenbein C, Chrousos G, Dacou-Voutetakis C (2013). Long-term clinical data and molecular defects in the STAR gene in five Greek patients. Eur. J. Endocrinol.

[45] Viswanath G, Chatterjee S, Dabral S, Nanguneri SR, Divya G, Roy P (2010). Anti-androgenic endocrine disrupting activities of chlorpyrifos and piperophos. J. Steroid Biochem. Mol. Biol.

[46] Stocco DM, Wang X, Jo Y, Manna PR (2005). Multiple signaling pathways regulating steroidogenesis and steroidogenic acute regulatory protein expression: more complicated than we thought. Mol. Endocrinol.

[47] Andrieu T, Pezzi V, Sirianni R, Le Bas R, Feral C, Benhaim A, Mittre H (2009). cAMP-dependent regulation of CYP19 gene in rabbit preovulatory granulosa cells and corpus luteum. J. Steroid Biochem. Mol. Biol.

[48] ElMazoudy RH, Attia AA (2012). Endocrine-disrupting and cytotoxic potential of anticholinesterase insecticide, diazinon in reproductive toxicity of male mice. J. Hazard. Mater.

[49] Verma R, Mohanty B (2009). Early-life exposure to dimethoate-induced reproductive toxicity: evaluation of effects on pituitary-testicular axis of mice. Toxicol. Sci.

[50] Aguilar-Garduno C, Lacasana M, Blanco-Munoz J, Rodriguez-Barranco M, Hernandez AF, Bassol S, Gonzalez-Alzaga B, Cebrian ME (2013). Changes in male hormone profile after occupational organophosphate exposure A longitudinal study. Toxicology.

[51] Bachelot A, Binart N (2005). Corpus luteum development: lessons from genetic models in mice. Curr. Top. Dev. Biol.

[52] Smallridge RC, Carr FE, Fein HG (1991). Diisopropylfluorophosphate (DFP) reduces serum prolactin, thyrotropin, luteinizing hormone, and growth hormone and increases adrenocorticotropin and corticosterone in rats: involvement of dopaminergic and somatostatinergic as well as cholinergic pathways. Toxicol. Appl. Pharmacol.

[53] Faro LR, Ferreira Nunes BV, Alfonso M, Ferreira VM, Duran R (2013). Role of glutamate receptors and nitric oxide on the effects of glufosinate ammonium, an organophosphate pesticide, on in-vivo dopamine release in rat striatum. Toxicology.

[54] Masoud A, Kiran R, Sandhir R (2011). Modulation of dopaminergic system and neurobehavioral functions in delayed neuropathy induced by organophosphates. Toxicol. Mech. Methods.

[55] Walsh LP, McCormick C, Martin C, Stocco DM (2000). Roundup inhibits steroidogenesis by disrupting steroidogenic acute regulatory (StAR) protein expression. Environ. Health Perspect.

